# (*E*)-*N*′-[2-(4-Chloro-3-nitro­phenyl­sulfon­yloxy)-3-methoxy­benzyl­idene]isonicotinohydrazide acetic acid tetra­solvate

**DOI:** 10.1107/S1600536808034089

**Published:** 2008-10-22

**Authors:** Xiao-Li Zhen, Xiao-Liu Li

**Affiliations:** a College of Chemistry & Environmental Science, Hebei University, Baoding 071002, People’s Republic of China; b College of Sciences, Hebei University of Science & Technology, Shijiazhuang 050018, People’s Republic of China

## Abstract

In the title compound, C_20_H_15_ClN_4_O_7_S·4CH_3_COOH, the central *o*-vanillin group makes dihedral angles of 9.50 (11) and 42.86 (7)°, respectively, with its attached pyridine and nitro­benzene rings. The crystal packing is stabilized by N—H⋯O, O—H⋯O and O—H⋯N hydrogen bonds and C—H⋯O inter­actions, leading to an infinite three-dimensional network. A short intramolecular C—H⋯O contact is also seen.

## Related literature

For general background, see: Allen *et al.* (1987 ▶); Jones *et al.* (1979 ▶); Larson & Pecoraro, (1991 ▶); Santos *et al.* (2001 ▶).
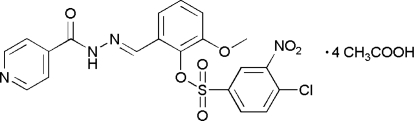


## Experimental

### 

#### Crystal data

C_20_H_15_ClN_4_O_7_S·4C_2_H_4_O_2_
*M* *_r_* = 731.09
Triclinic, 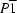

*a* = 8.0565 (16) Å
*b* = 13.876 (3) Å
*c* = 16.097 (3) Å
α = 79.01 (3)°
β = 76.01 (3)°
γ = 75.44 (3)°
*V* = 1673.8 (7) Å^3^
*Z* = 2
Mo *K*α radiation
μ = 0.25 mm^−1^
*T* = 294 (2) K
0.23 × 0.18 × 0.12 mm

#### Data collection

Bruker SMART APEX CCD area-detector diffractometer
Absorption correction: multi-scan (*SADABS*; Sheldrick, 1996 ▶) *T*_min_ = 0.913, *T*_max_ = 0.970
9794 measured reflections
5882 independent reflections
4448 reflections with *I* > 2σ(*I*)
*R*_int_ = 0.081

#### Refinement

*R*[*F* ^2^ > 2σ(*F* ^2^)] = 0.059
*wR*(*F* ^2^) = 0.146
*S* = 1.01
5882 reflections
451 parameters
H-atom parameters constrained
Δρ_max_ = 0.30 e Å^−3^
Δρ_min_ = −0.44 e Å^−3^

### 

Data collection: *SMART* (Bruker, 1999 ▶); cell refinement: *SAINT* (Bruker, 1999 ▶); data reduction: *SAINT*; program(s) used to solve structure: *SHELXS97* (Sheldrick, 2008 ▶); program(s) used to refine structure: *SHELXL97* (Sheldrick, 2008 ▶); molecular graphics: *PLATON* (Spek, 2003 ▶); software used to prepare material for publication: *SHELXTL* (Sheldrick, 2008 ▶).

## Supplementary Material

Crystal structure: contains datablocks I, global. DOI: 10.1107/S1600536808034089/at2646sup1.cif

Structure factors: contains datablocks I. DOI: 10.1107/S1600536808034089/at2646Isup2.hkl

Additional supplementary materials:  crystallographic information; 3D view; checkCIF report

## Figures and Tables

**Table 1:** Hydrogen-bond geometry (Å, °)

*D*—H⋯*A*	*D*—H	H⋯*A*	*D*⋯*A*	*D*—H⋯*A*
O71—H71⋯N11^i^	0.82	1.84	2.661 (3)	176
C35—H35⋯O17^ii^	0.93	2.51	3.360 (3)	152
O41—H41⋯O17^iii^	0.82	1.96	2.698 (3)	150
O41—H41⋯N27^iii^	0.82	2.59	3.211 (3)	134
N17—H17⋯O42^iv^	0.86	2.10	2.875 (3)	150
C27—H27⋯O42^iv^	0.93	2.43	3.206 (4)	140
O51—H51⋯O52^iii^	0.82	1.87	2.672 (3)	167
C24—H24⋯O62^iii^	0.93	2.54	3.461 (3)	170
O61—H61⋯O62^v^	0.82	1.83	2.648 (3)	178
C12—H12⋯O72^i^	0.93	2.59	3.255 (3)	129
C32—H32⋯O72	0.93	2.37	3.122 (4)	137

Symmetry codes: (i) 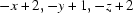; (ii) 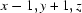; (iii) 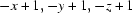; (iv) 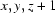; (v) 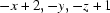.

**Fig. 1.**
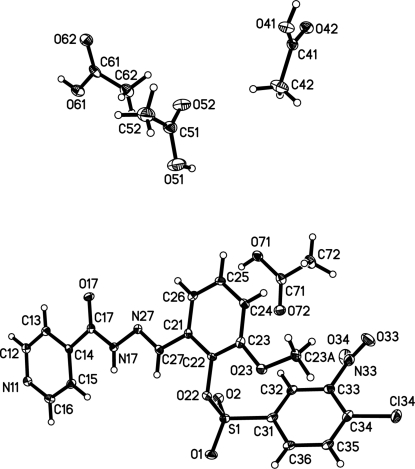
The structure of (I) with displacement ellipsoids for non-H atoms drawn at the 30% probability level.

**Fig. 2.**
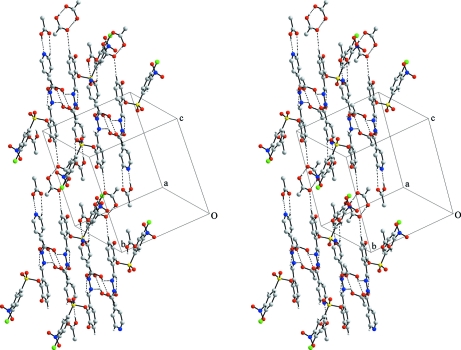
A stereoview of part of the crystal structure of compound (I), showing the formation of a complex network. For the sake of clarity, H atoms not involved in the hydrogen bonds shown have been omitted.

## supplementary crystallographic information

### Comment

There has been a steady growth of interest in the synthesis, structure, and reactivity of Schiff bases due to their potential applications in areas such as biological modelling, catalysis, and molecular magnets (Jones *et al.*, 1979; Larson & Pecoraro, 1991). One of the aims of investigating the structural chemistry of Schiff bases is to develop protein and enzyme mimics (Santos *et al.*, 2001). As part of an investigation of the coordination properties of Schiff bases functioning as ligands, we report the synthesis and structure of the title compound (I).

In the title molecule (Fig. 1), bond lengths and angles are within normal ranges (Allen *et al.*, 1987). The *o*-vanillin group (C21–C27/O22/O23) is essentially planar, with an r.m.s. deviation for fitted atoms of 0.0236 Å. This group makes dihedral angles of 9.50 (11)° and 42.86 (7)°, respectively, with the pyridine ring (C12—C16/N11) and the benzene ring (C31—C36). Furthermore, the dihedral angle between the pyridine ring (C12—C16/N11) and the benzene ring (C31—C36) is 50.43 (8)°.

The crystal packing is stabilized by N—H···O, O—H···O, O—H···N hydrogen bonds and C—H···O interactions (Table 1, Fig. 2), leading to an infinite network.

### Experimental

An anhydrous ethanol solution (50 ml) of 2-formyl-6-methoxyphenyl 4-chloro-3-nitrobenzenesulfonate (3.72 g, 10 mmol) was added to an anhydrous ethanol solution (50 ml) of isonicotinohydrazide (1.37 g, 10 mmol) and the mixture stirred at 350 K for 5 h under nitrogen, giving a yellow precipitate. The product was isolated, recrystallized from ethanol and then dried in a vacuum to give the pure compound in 78% yield. Yellow single crystals of (I) suitable for X-ray analysis were obtained by slow evaporation of an acetic acid solution.

### Refinement

The H atoms of the water molecule and imine group were located in difference maps and then treated as riding atoms. All other H atoms were included in calculated positions and refined using a riding model approximation. Constrained C—H, O—H and N—H bond lengths and isotropic U parameters: 0.93 Å and *U*_iso_(H) = 1.2*U*_eq_(C) for C*sp*^2^—H; 0.96 Å and *U*_iso_(H) = 1.5*U*_eq_(C) for methyl C—H; 0.82 Å and *U*_iso_(H) = 1.5*U*_eq_(O) for hydroxyl O—H; 0.86 Å and *U*_iso_(H) = 1.2*U*_eq_(N) for imino N—H.

### Crystal data

**Table:**

C_20_H_15_ClN_4_O_7_S·4C_2_H_4_O_2_	*Z* = 2
*M**_r_* = 731.09	*F*(000) = 760
Triclinic, *P*1	*D*_x_ = 1.451 Mg m^−^^3^
Hall symbol: -P 1	Mo *K*α radiation, λ = 0.71073 Å
*a* = 8.0565 (16) Å	Cell parameters from 4854 reflections
*b* = 13.876 (3) Å	θ = 2.2–28.0°
*c* = 16.097 (3) Å	µ = 0.25 mm^−^^1^
α = 79.01 (3)°	*T* = 294 K
β = 76.01 (3)°	Block, yellow
γ = 75.44 (3)°	0.23 × 0.18 × 0.12 mm
*V* = 1673.8 (7) Å^3^	

### Data collection

**Table:**

Bruker SMART APEX CCD area-detector diffractometer	5882 independent reflections
Radiation source: fine-focus sealed tube	4448 reflections with *I* > 2σ(*I*)
graphite	*R*_int_ = 0.081
φ and ω scans	θ_max_ = 25.0°, θ_min_ = 1.9°
Absorption correction: multi-scan (SADABS; Sheldrick, 1996)	*h* = −8→9
*T*_min_ = 0.913, *T*_max_ = 0.970	*k* = −16→13
9794 measured reflections	*l* = −19→14

### Refinement

**Table:**

Refinement on *F*^2^	Primary atom site location: structure-invariant direct methods
Least-squares matrix: full	Secondary atom site location: difference Fourier map
*R*[*F*^2^ > 2σ(*F*^2^)] = 0.059	Hydrogen site location: inferred from neighbouring sites
*wR*(*F*^2^) = 0.146	H-atom parameters constrained
*S* = 1.01	*w* = 1/[σ^2^(*F*_o_^2^) + (0.0627*P*)^2^] where *P* = (*F*_o_^2^ + 2*F*_c_^2^)/3
5882 reflections	(Δ/σ)_max_ = 0.001
451 parameters	Δρ_max_ = 0.30 e Å^−^^3^
0 restraints	Δρ_min_ = −0.44 e Å^−^^3^

### Special details

**Table:**

Geometry. All e.s.d.'s (except the e.s.d. in the dihedral angle between two l.s. planes) are estimated using the full covariance matrix. The cell e.s.d.'s are taken into account individually in the estimation of e.s.d.'s in distances, angles and torsion angles; correlations between e.s.d.'s in cell parameters are only used when they are defined by crystal symmetry. An approximate (isotropic) treatment of cell e.s.d.'s is used for estimating e.s.d.'s involving l.s. planes.
Refinement. Refinement of *F*^2^ against ALL reflections. The weighted *R*-factor *wR* and goodness of fit *S* are based on *F*^2^, conventional *R*-factors *R* are based on *F*, with *F* set to zero for negative *F*^2^. The threshold expression of *F*^2^ > 2σ(*F*^2^) is used only for calculating *R*-factors(gt) *etc*. and is not relevant to the choice of reflections for refinement. *R*-factors based on *F*^2^ are statistically about twice as large as those based on *F*, and *R*- factors based on ALL data will be even larger.

### Fractional atomic coordinates and isotropic or equivalent isotropic displacement parameters (Å^2^)

**Table:**

	*x*	*y*	*z*	*U*_iso_*/*U*_eq_	
S1	0.36674 (7)	0.89584 (4)	0.99002 (5)	0.01955 (19)	
O1	0.2825 (2)	0.90760 (14)	1.07606 (14)	0.0286 (5)	
O2	0.5507 (2)	0.85500 (13)	0.96749 (14)	0.0244 (5)	
N11	1.0675 (2)	0.32110 (15)	1.23406 (16)	0.0208 (5)	
C12	1.1069 (3)	0.27264 (19)	1.1657 (2)	0.0207 (6)	
H12	1.1880	0.2117	1.1662	0.025*	
C13	1.0324 (3)	0.30910 (18)	1.0941 (2)	0.0201 (6)	
H13	1.0630	0.2731	1.0475	0.024*	
C14	0.9115 (3)	0.40008 (17)	1.09249 (18)	0.0169 (6)	
C15	0.8746 (3)	0.45215 (19)	1.1617 (2)	0.0226 (6)	
H15	0.7976	0.5146	1.1618	0.027*	
C16	0.9547 (3)	0.4098 (2)	1.2318 (2)	0.0247 (6)	
H16	0.9284	0.4448	1.2788	0.030*	
C17	0.8325 (3)	0.43673 (18)	1.01316 (19)	0.0178 (6)	
N17	0.7134 (2)	0.52433 (14)	1.01405 (16)	0.0181 (5)	
H17	0.6802	0.5543	1.0593	0.022*	
O17	0.8782 (2)	0.38954 (13)	0.95161 (13)	0.0238 (5)	
C21	0.4519 (3)	0.70078 (17)	0.87464 (18)	0.0164 (6)	
C22	0.3222 (3)	0.78844 (18)	0.88440 (18)	0.0155 (5)	
O22	0.26276 (19)	0.82421 (12)	0.96469 (12)	0.0177 (4)	
C23	0.2359 (3)	0.83875 (17)	0.81745 (19)	0.0189 (6)	
O23	0.1050 (2)	0.91963 (12)	0.83710 (13)	0.0214 (4)	
C23A	0.0280 (3)	0.9805 (2)	0.7665 (2)	0.0268 (7)	
H23A	−0.0291	0.9413	0.7440	0.040*	
H23B	0.1183	1.0029	0.7216	0.040*	
H23C	−0.0561	1.0378	0.7871	0.040*	
C24	0.2833 (3)	0.80131 (18)	0.73985 (19)	0.0189 (6)	
H24	0.2276	0.8335	0.6946	0.023*	
C25	0.4150 (3)	0.71516 (18)	0.72938 (19)	0.0200 (6)	
H25	0.4475	0.6907	0.6765	0.024*	
C26	0.4983 (3)	0.66517 (18)	0.7960 (2)	0.0206 (6)	
H26	0.5857	0.6075	0.7878	0.025*	
C27	0.5316 (3)	0.64754 (17)	0.94872 (19)	0.0178 (6)	
H27	0.4993	0.6737	1.0008	0.021*	
N27	0.6456 (2)	0.56518 (14)	0.94087 (15)	0.0173 (5)	
C31	0.3174 (3)	1.00961 (18)	0.92192 (19)	0.0202 (6)	
C32	0.4306 (3)	1.02540 (18)	0.8445 (2)	0.0210 (6)	
H32	0.5329	0.9781	0.8287	0.025*	
C33	0.3873 (3)	1.11389 (19)	0.7910 (2)	0.0230 (6)	
N33	0.5045 (3)	1.13006 (17)	0.7079 (2)	0.0342 (6)	
O33	0.4431 (3)	1.14733 (18)	0.64263 (17)	0.0503 (7)	
O34	0.6588 (2)	1.12465 (17)	0.70740 (18)	0.0486 (7)	
C34	0.2382 (3)	1.18656 (19)	0.8158 (2)	0.0246 (7)	
Cl34	0.19242 (9)	1.30033 (5)	0.75118 (6)	0.0351 (2)	
C35	0.1276 (3)	1.16966 (19)	0.8944 (2)	0.0244 (6)	
H35	0.0273	1.2179	0.9111	0.029*	
C36	0.1665 (3)	1.08062 (19)	0.9483 (2)	0.0223 (6)	
H36	0.0928	1.0682	1.0015	0.027*	
C41	0.3533 (3)	0.61046 (19)	0.2126 (2)	0.0254 (7)	
O41	0.2137 (2)	0.58404 (16)	0.20296 (15)	0.0329 (5)	
H41	0.2225	0.5796	0.1519	0.049*	
O42	0.4795 (2)	0.61927 (15)	0.15511 (15)	0.0318 (5)	
C42	0.3373 (4)	0.6290 (3)	0.3024 (2)	0.0473 (9)	
H42A	0.4483	0.6360	0.3097	0.071*	
H42B	0.3020	0.5735	0.3424	0.071*	
H42C	0.2512	0.6896	0.3131	0.071*	
C51	0.3163 (4)	0.4268 (2)	0.5144 (2)	0.0378 (8)	
O51	0.2940 (3)	0.4842 (2)	0.57278 (19)	0.0548 (7)	
H51	0.3672	0.5192	0.5583	0.082*	
O52	0.4437 (3)	0.42050 (18)	0.45479 (18)	0.0491 (6)	
C52	0.1786 (5)	0.3693 (3)	0.5263 (3)	0.0520 (10)	
H52A	0.1674	0.3586	0.4709	0.078*	
H52B	0.0691	0.4065	0.5549	0.078*	
H52C	0.2098	0.3056	0.5608	0.078*	
C61	0.8864 (3)	0.1393 (2)	0.4933 (2)	0.0299 (7)	
O61	0.9839 (2)	0.10855 (16)	0.55199 (15)	0.0348 (5)	
H61	1.0288	0.0486	0.5514	0.052*	
O62	0.8689 (3)	0.08503 (15)	0.44639 (16)	0.0371 (6)	
C62	0.7966 (4)	0.2472 (2)	0.4893 (2)	0.0400 (8)	
H62A	0.6736	0.2527	0.5125	0.060*	
H62B	0.8137	0.2787	0.4302	0.060*	
H62C	0.8446	0.2799	0.5225	0.060*	
C71	0.6959 (3)	0.8590 (2)	0.6418 (2)	0.0239 (7)	
O71	0.7975 (2)	0.77024 (14)	0.62854 (14)	0.0272 (5)	
H71	0.8338	0.7429	0.6725	0.041*	
O72	0.6782 (2)	0.89620 (14)	0.70593 (15)	0.0316 (5)	
C72	0.6031 (3)	0.9089 (2)	0.5696 (2)	0.0333 (8)	
H72A	0.4879	0.8950	0.5829	0.050*	
H72B	0.6679	0.8834	0.5171	0.050*	
H72C	0.5940	0.9801	0.5626	0.050*	

### Atomic displacement parameters (Å^2^)

**Table:**

	*U*^11^	*U*^22^	*U*^33^	*U*^12^	*U*^13^	*U*^23^
S1	0.0179 (3)	0.0224 (4)	0.0203 (4)	−0.0036 (2)	−0.0060 (3)	−0.0059 (3)
O1	0.0352 (10)	0.0325 (11)	0.0204 (13)	−0.0055 (8)	−0.0070 (9)	−0.0096 (9)
O2	0.0186 (8)	0.0266 (10)	0.0302 (13)	−0.0039 (7)	−0.0092 (8)	−0.0054 (9)
N11	0.0165 (10)	0.0231 (12)	0.0227 (15)	−0.0043 (8)	−0.0057 (10)	−0.0009 (11)
C12	0.0142 (11)	0.0227 (14)	0.0237 (18)	−0.0045 (9)	−0.0026 (11)	−0.0008 (12)
C13	0.0182 (11)	0.0201 (13)	0.0230 (17)	−0.0059 (9)	−0.0039 (11)	−0.0033 (12)
C14	0.0149 (11)	0.0201 (13)	0.0157 (16)	−0.0073 (9)	−0.0026 (11)	0.0013 (11)
C15	0.0225 (12)	0.0190 (13)	0.0261 (18)	−0.0003 (10)	−0.0083 (12)	−0.0041 (12)
C16	0.0236 (12)	0.0305 (15)	0.0203 (18)	−0.0041 (11)	−0.0049 (12)	−0.0059 (13)
C17	0.0151 (11)	0.0184 (13)	0.0208 (17)	−0.0075 (10)	−0.0030 (11)	−0.0002 (12)
N17	0.0182 (9)	0.0173 (11)	0.0195 (14)	−0.0022 (8)	−0.0068 (10)	−0.0027 (10)
O17	0.0265 (9)	0.0212 (10)	0.0237 (13)	0.0021 (7)	−0.0087 (9)	−0.0081 (9)
C21	0.0148 (10)	0.0163 (13)	0.0191 (16)	−0.0064 (9)	−0.0043 (11)	0.0006 (11)
C22	0.0154 (11)	0.0199 (13)	0.0123 (15)	−0.0068 (9)	−0.0007 (10)	−0.0037 (11)
O22	0.0166 (8)	0.0209 (9)	0.0157 (11)	−0.0048 (6)	−0.0028 (8)	−0.0028 (8)
C23	0.0140 (11)	0.0165 (13)	0.0275 (18)	−0.0048 (9)	−0.0065 (11)	−0.0015 (12)
O23	0.0190 (8)	0.0243 (9)	0.0199 (12)	0.0013 (7)	−0.0076 (8)	−0.0036 (8)
C23A	0.0271 (13)	0.0265 (14)	0.0245 (19)	0.0019 (11)	−0.0139 (13)	0.0029 (13)
C24	0.0189 (11)	0.0238 (14)	0.0166 (16)	−0.0087 (10)	−0.0057 (11)	−0.0011 (12)
C25	0.0217 (12)	0.0239 (14)	0.0167 (16)	−0.0080 (10)	−0.0020 (11)	−0.0064 (12)
C26	0.0182 (11)	0.0181 (13)	0.0266 (18)	−0.0037 (10)	−0.0043 (12)	−0.0063 (12)
C27	0.0175 (11)	0.0206 (13)	0.0164 (16)	−0.0079 (10)	−0.0035 (11)	−0.0002 (11)
N27	0.0161 (9)	0.0174 (11)	0.0194 (14)	−0.0043 (8)	−0.0063 (9)	−0.0008 (9)
C31	0.0165 (11)	0.0208 (13)	0.0270 (18)	−0.0056 (9)	−0.0048 (12)	−0.0099 (12)
C32	0.0156 (11)	0.0191 (13)	0.0281 (18)	0.0008 (9)	−0.0048 (12)	−0.0080 (12)
C33	0.0201 (12)	0.0254 (14)	0.0232 (18)	−0.0062 (10)	0.0001 (12)	−0.0068 (12)
N33	0.0341 (13)	0.0245 (13)	0.0343 (19)	−0.0002 (10)	0.0037 (12)	−0.0027 (12)
O33	0.0529 (13)	0.0589 (15)	0.0228 (15)	0.0053 (11)	0.0007 (12)	−0.0003 (12)
O34	0.0249 (10)	0.0518 (14)	0.0540 (19)	−0.0077 (9)	0.0101 (11)	0.0052 (12)
C34	0.0211 (12)	0.0189 (13)	0.036 (2)	−0.0016 (10)	−0.0101 (13)	−0.0067 (12)
Cl34	0.0359 (4)	0.0236 (4)	0.0399 (5)	0.0018 (3)	−0.0083 (4)	−0.0007 (3)
C35	0.0187 (12)	0.0229 (14)	0.0305 (19)	−0.0001 (10)	−0.0027 (12)	−0.0102 (13)
C36	0.0162 (11)	0.0262 (14)	0.0263 (18)	−0.0056 (10)	−0.0008 (11)	−0.0112 (12)
C41	0.0258 (13)	0.0254 (14)	0.0229 (19)	−0.0002 (11)	−0.0073 (13)	−0.0027 (13)
O41	0.0276 (10)	0.0474 (13)	0.0254 (14)	−0.0059 (8)	−0.0047 (10)	−0.0127 (11)
O42	0.0275 (10)	0.0381 (11)	0.0261 (14)	−0.0023 (8)	−0.0008 (10)	−0.0080 (10)
C42	0.0498 (19)	0.073 (2)	0.027 (2)	−0.0286 (17)	−0.0041 (17)	−0.0110 (19)
C51	0.0457 (18)	0.0357 (18)	0.032 (2)	0.0015 (14)	−0.0159 (17)	−0.0072 (16)
O51	0.0612 (15)	0.0578 (16)	0.050 (2)	−0.0169 (12)	0.0004 (14)	−0.0288 (15)
O52	0.0518 (14)	0.0538 (15)	0.0441 (18)	−0.0069 (11)	−0.0038 (13)	−0.0264 (13)
C52	0.059 (2)	0.061 (2)	0.042 (3)	−0.0182 (17)	−0.0104 (19)	−0.0138 (19)
C61	0.0215 (13)	0.0445 (18)	0.0185 (18)	−0.0082 (12)	0.0015 (12)	0.0021 (15)
O61	0.0312 (10)	0.0453 (12)	0.0282 (14)	−0.0054 (9)	−0.0093 (10)	−0.0054 (10)
O62	0.0427 (12)	0.0388 (12)	0.0330 (15)	−0.0062 (9)	−0.0180 (11)	−0.0033 (11)
C62	0.0346 (15)	0.0464 (19)	0.034 (2)	−0.0027 (13)	−0.0053 (15)	−0.0036 (16)
C71	0.0169 (12)	0.0291 (16)	0.0236 (19)	−0.0072 (11)	−0.0007 (12)	0.0002 (13)
O71	0.0268 (9)	0.0301 (11)	0.0226 (13)	0.0001 (8)	−0.0089 (9)	−0.0017 (9)
O72	0.0359 (10)	0.0298 (11)	0.0283 (14)	0.0003 (8)	−0.0091 (10)	−0.0086 (10)
C72	0.0278 (14)	0.0384 (17)	0.029 (2)	−0.0028 (12)	−0.0090 (14)	0.0035 (14)

### Geometric parameters (Å, °)

**Table:**

S1—O1	1.408 (2)	C32—C33	1.378 (4)
S1—O2	1.4303 (18)	C32—H32	0.9300
S1—O22	1.6107 (17)	C33—C34	1.390 (4)
S1—C31	1.757 (3)	C33—N33	1.453 (4)
N11—C12	1.330 (4)	N33—O33	1.225 (4)
N11—C16	1.334 (3)	N33—O34	1.225 (3)
C12—C13	1.380 (4)	C34—C35	1.377 (4)
C12—H12	0.9300	C34—Cl34	1.726 (3)
C13—C14	1.388 (3)	C35—C36	1.380 (4)
C13—H13	0.9300	C35—H35	0.9300
C14—C15	1.375 (4)	C36—H36	0.9300
C14—C17	1.509 (4)	C41—O42	1.212 (3)
C15—C16	1.393 (4)	C41—O41	1.318 (3)
C15—H15	0.9300	C41—C42	1.486 (5)
C16—H16	0.9300	O41—H41	0.8200
C17—O17	1.223 (3)	C42—H42A	0.9600
C17—N17	1.346 (3)	C42—H42B	0.9600
N17—N27	1.381 (3)	C42—H42C	0.9600
N17—H17	0.8600	C51—O52	1.222 (4)
C21—C26	1.380 (4)	C51—O51	1.297 (4)
C21—C22	1.397 (3)	C51—C52	1.479 (5)
C21—C27	1.475 (3)	O51—H51	0.8200
C22—C23	1.401 (3)	C52—H52A	0.9600
C22—O22	1.404 (3)	C52—H52B	0.9600
C23—O23	1.360 (3)	C52—H52C	0.9600
C23—C24	1.376 (4)	C61—O62	1.216 (4)
O23—C23A	1.450 (3)	C61—O61	1.315 (3)
C23A—H23A	0.9600	C61—C62	1.487 (4)
C23A—H23B	0.9600	O61—H61	0.8200
C23A—H23C	0.9600	C62—H62A	0.9600
C24—C25	1.391 (4)	C62—H62B	0.9600
C24—H24	0.9300	C62—H62C	0.9600
C25—C26	1.383 (4)	C71—O72	1.203 (4)
C25—H25	0.9300	C71—O71	1.320 (3)
C26—H26	0.9300	C71—C72	1.497 (4)
C27—N27	1.279 (3)	O71—H71	0.8200
C27—H27	0.9300	C72—H72A	0.9600
C31—C32	1.371 (4)	C72—H72B	0.9600
C31—C36	1.392 (4)	C72—H72C	0.9600
			
O1—S1—O2	121.40 (12)	C36—C31—S1	119.3 (2)
O1—S1—O22	103.04 (11)	C31—C32—C33	117.6 (2)
O2—S1—O22	108.77 (10)	C31—C32—H32	121.2
O1—S1—C31	110.02 (13)	C33—C32—H32	121.2
O2—S1—C31	108.32 (12)	C32—C33—C34	121.4 (3)
O22—S1—C31	103.83 (10)	C32—C33—N33	117.9 (2)
C12—N11—C16	118.2 (2)	C34—C33—N33	120.7 (2)
N11—C12—C13	122.7 (2)	O33—N33—O34	124.1 (3)
N11—C12—H12	118.6	O33—N33—C33	118.0 (2)
C13—C12—H12	118.6	O34—N33—C33	117.9 (3)
C12—C13—C14	119.2 (3)	C35—C34—C33	120.0 (2)
C12—C13—H13	120.4	C35—C34—Cl34	119.0 (2)
C14—C13—H13	120.4	C33—C34—Cl34	121.0 (2)
C15—C14—C13	118.3 (2)	C34—C35—C36	119.6 (2)
C15—C14—C17	124.2 (2)	C34—C35—H35	120.2
C13—C14—C17	117.4 (3)	C36—C35—H35	120.2
C14—C15—C16	118.9 (2)	C35—C36—C31	119.1 (3)
C14—C15—H15	120.6	C35—C36—H36	120.4
C16—C15—H15	120.6	C31—C36—H36	120.4
N11—C16—C15	122.6 (3)	O42—C41—O41	124.2 (3)
N11—C16—H16	118.7	O42—C41—C42	123.9 (2)
C15—C16—H16	118.7	O41—C41—C42	111.9 (3)
O17—C17—N17	122.8 (2)	C41—O41—H41	109.5
O17—C17—C14	121.1 (2)	C41—C42—H42A	109.5
N17—C17—C14	116.1 (3)	C41—C42—H42B	109.5
C17—N17—N27	118.7 (2)	H42A—C42—H42B	109.5
C17—N17—H17	120.7	C41—C42—H42C	109.5
N27—N17—H17	120.7	H42A—C42—H42C	109.5
C26—C21—C22	118.6 (2)	H42B—C42—H42C	109.5
C26—C21—C27	121.9 (2)	O52—C51—O51	121.7 (3)
C22—C21—C27	119.5 (3)	O52—C51—C52	123.1 (3)
C21—C22—C23	121.7 (3)	O51—C51—C52	115.2 (3)
C21—C22—O22	120.7 (2)	C51—O51—H51	109.5
C23—C22—O22	117.4 (2)	C51—C52—H52A	109.5
C22—O22—S1	119.17 (15)	C51—C52—H52B	109.5
O23—C23—C24	125.5 (2)	H52A—C52—H52B	109.5
O23—C23—C22	115.7 (3)	C51—C52—H52C	109.5
C24—C23—C22	118.7 (2)	H52A—C52—H52C	109.5
C23—O23—C23A	116.9 (2)	H52B—C52—H52C	109.5
O23—C23A—H23A	109.5	O62—C61—O61	123.7 (3)
O23—C23A—H23B	109.5	O62—C61—C62	122.3 (3)
H23A—C23A—H23B	109.5	O61—C61—C62	114.1 (3)
O23—C23A—H23C	109.5	C61—O61—H61	109.5
H23A—C23A—H23C	109.5	C61—C62—H62A	109.5
H23B—C23A—H23C	109.5	C61—C62—H62B	109.5
C23—C24—C25	119.7 (2)	H62A—C62—H62B	109.5
C23—C24—H24	120.1	C61—C62—H62C	109.5
C25—C24—H24	120.1	H62A—C62—H62C	109.5
C26—C25—C24	121.3 (3)	H62B—C62—H62C	109.5
C26—C25—H25	119.3	O72—C71—O71	123.4 (2)
C24—C25—H25	119.3	O72—C71—C72	123.0 (3)
C21—C26—C25	120.0 (2)	O71—C71—C72	113.5 (3)
C21—C26—H26	120.0	C71—O71—H71	109.5
C25—C26—H26	120.0	C71—C72—H72A	109.5
N27—C27—C21	119.6 (3)	C71—C72—H72B	109.5
N27—C27—H27	120.2	H72A—C72—H72B	109.5
C21—C27—H27	120.2	C71—C72—H72C	109.5
C27—N27—N17	113.8 (2)	H72A—C72—H72C	109.5
C32—C31—C36	122.3 (2)	H72B—C72—H72C	109.5
C32—C31—S1	118.43 (19)		
			
C16—N11—C12—C13	−1.8 (3)	C23—C24—C25—C26	0.8 (3)
N11—C12—C13—C14	0.1 (4)	C22—C21—C26—C25	−1.0 (3)
C12—C13—C14—C15	2.1 (3)	C27—C21—C26—C25	177.3 (2)
C12—C13—C14—C17	−179.6 (2)	C24—C25—C26—C21	−0.3 (3)
C13—C14—C15—C16	−2.5 (3)	C26—C21—C27—N27	−1.0 (3)
C17—C14—C15—C16	179.3 (2)	C22—C21—C27—N27	177.3 (2)
C12—N11—C16—C15	1.4 (4)	C21—C27—N27—N17	178.38 (18)
C14—C15—C16—N11	0.8 (4)	C17—N17—N27—C27	179.00 (19)
C15—C14—C17—O17	175.8 (2)	O1—S1—C31—C32	156.83 (19)
C13—C14—C17—O17	−2.4 (3)	O2—S1—C31—C32	22.0 (2)
C15—C14—C17—N17	−2.9 (3)	O22—S1—C31—C32	−93.5 (2)
C13—C14—C17—N17	178.9 (2)	O1—S1—C31—C36	−22.8 (2)
O17—C17—N17—N27	−3.1 (3)	O2—S1—C31—C36	−157.57 (19)
C14—C17—N17—N27	175.57 (18)	O22—S1—C31—C36	86.9 (2)
C26—C21—C22—C23	1.8 (3)	C36—C31—C32—C33	−2.1 (4)
C27—C21—C22—C23	−176.5 (2)	S1—C31—C32—C33	178.31 (18)
C26—C21—C22—O22	176.0 (2)	C31—C32—C33—C34	2.6 (4)
C27—C21—C22—O22	−2.3 (3)	C31—C32—C33—N33	−178.5 (2)
C21—C22—O22—S1	86.2 (2)	C32—C33—N33—O33	123.9 (3)
C23—C22—O22—S1	−99.4 (2)	C34—C33—N33—O33	−57.3 (4)
O1—S1—O22—C22	−174.26 (16)	C32—C33—N33—O34	−55.5 (3)
O2—S1—O22—C22	−44.21 (18)	C34—C33—N33—O34	123.4 (3)
C31—S1—O22—C22	70.97 (19)	C32—C33—C34—C35	−1.9 (4)
C21—C22—C23—O23	176.15 (19)	N33—C33—C34—C35	179.3 (2)
O22—C22—C23—O23	1.7 (3)	C32—C33—C34—Cl34	175.97 (19)
C21—C22—C23—C24	−1.2 (3)	N33—C33—C34—Cl34	−2.9 (4)
O22—C22—C23—C24	−175.7 (2)	C33—C34—C35—C36	0.5 (4)
C24—C23—O23—C23A	−10.3 (3)	Cl34—C34—C35—C36	−177.41 (19)
C22—C23—O23—C23A	172.6 (2)	C34—C35—C36—C31	0.1 (4)
O23—C23—C24—C25	−177.2 (2)	C32—C31—C36—C35	0.8 (4)
C22—C23—C24—C25	−0.1 (3)	S1—C31—C36—C35	−179.63 (18)

### Hydrogen-bond geometry (Å, °)

**Table:**

*D*—H···*A*	*D*—H	H···*A*	*D*···*A*	*D*—H···*A*
O71—H71···N11^i^	0.82	1.84	2.661 (3)	176
C35—H35···O17^ii^	0.93	2.51	3.360 (3)	152
O41—H41···O17^iii^	0.82	1.96	2.698 (3)	150
O41—H41···N27^iii^	0.82	2.59	3.211 (3)	134
N17—H17···O42^iv^	0.86	2.10	2.875 (3)	150
C27—H27···O42^iv^	0.93	2.43	3.206 (4)	140
O51—H51···O52^iii^	0.82	1.87	2.672 (3)	167
C24—H24···O62^iii^	0.93	2.54	3.461 (3)	170
O61—H61···O62^v^	0.82	1.83	2.648 (3)	178
C12—H12···O72^i^	0.93	2.59	3.255 (3)	129
C32—H32···O72	0.93	2.37	3.122 (4)	137

Symmetry codes: (i) −*x*+2, −*y*+1, −*z*+2; (ii) *x*−1, *y*+1, *z*; (iii) −*x*+1, −*y*+1, −*z*+1; (iv) *x*, *y*, *z*+1; (v) −*x*+2, −*y*, −*z*+1.
